# Mastering targeted genome engineering of GC-rich oleaginous yeast for tailored plant oil alternatives for the food and chemical sector

**DOI:** 10.1186/s12934-023-02033-1

**Published:** 2023-02-08

**Authors:** Pariya Shaigani, Tobias Fuchs, Petra Graban, Sophia Prem, Martina Haack, Mahmoud Masri, Norbert Mehlmer, Thomas Brueck

**Affiliations:** Department of Chemistry, Werner Siemens-Chair of Synthetic Biotechnology, Technical University of Munich, Garching, Germany

**Keywords:** Oleaginous yeast, Yeast oil, Genome engineering, CRISPR/Cas, Tailored plant oil alternatives, *Cutaneotrichosporon oleaginosus*, High-oleic sunflower oil, Cocoa butter, Fatty acid biosynthesis

## Abstract

**Background:**

Sustainable production of triglycerides for various applications is a major focus of microbial factories. Oleaginous yeast species have been targeted for commercial production of microbial oils. Among all the oleaginous yeasts examined in a previous comparative study, *Cutaneotrichosporon oleaginosus* showed the highest lipid productivity. Moreover, a new lipid production process for *C. oleaginosus* with minimal waste generation and energy consumption resulted in the highest lipid productivity in the history of oleaginous yeasts. However, productivity and product diversity are restricted because of the genetic intractability of this yeast. To date, successful targeted genetic engineering of *C. oleaginosus* has not yet been reported.

**Results:**

The targeted gene editing was successfully carried out in *C. oleaginosus* using CRISPR/Cas system. A tailored enzyme system isolated to degrade the *C. oleaginosus* cell wall enabled the isolation of viable spheroplasts that are amenable to in-cell delivery of nucleic acids and proteins. The employment of both Cas9 protein and Cas mRNA was effective in obtaining strains with *URA5* knockout that did not exhibit growth in the absence of uracil. Subsequently, we successfully created several strains with enhanced lipid yield (54% increase compared to that in wild type) or modified fatty acid profiles comparable with those of cocoa butter or sunflower oil compositions.

**Conclusion:**

This study establishes the first targeted engineering technique for *C. oleaginosus* using the CRISPR/Cas system. The current study creates the foundation for flexible and targeted strain optimizations towards building a robust platform for sustainable microbial lipid production. Moreover, the genetic transformation of eukaryotic microbial cells using Cas9 mRNA was successfully achieved.

**Supplementary Information:**

The online version contains supplementary material available at 10.1186/s12934-023-02033-1.

## Background

The increasing global demand for plant- and animal-based lipids in the biofuel, pharmaceutical, and oleochemical industries has negatively impacted biodiversity and caused land-use changes. Microbial oils have shown significant improvements in yield and sustainability compared to vegetable oils [1–4]. The oleaginous yeast *Cutaneotrichosporon oleaginosus* can accumulate lipids via the *de-novo* lipid biosynthesis pathway. Its superiority over other oleaginous microorganisms in having high triglyceride (TAG) content, substrate flexibility, and rapid growth rate has made this yeast a prime candidate for oleochemical production [5–7]. To date, C. *oleaginosus* has displayed the highest carbon source flexibility linked with high *de-novo* oil productivity and yield of any oleaginous yeast strain [7–13]. Moreover, compared to other yeasts, *C. oleaginosus* is highly resistant to fermentation inhibitors (including weak organic acids e.g. acetic acid [8], sugar-derived furans, and phenolic compounds [14]) formed during hydrolysis and pre-treatment processes. This enables the cultivation of *C. oleaginosus* in cost-efficient, undetoxified, and complex waste biomass streams. This economic potential of *C. oleaginosus* has led to a great interest in generating tailor-made lipids for the large-scale production of various high-value fats [15]. The cost of microbial oil production is higher than that of the common plant oils [8, 15]; however, in a previous study, a highly efficient lipid production process by *C. oleaginosus* using a new, residue-free, circular production process with minimal energy consumption was established that reached a maximum of 2.4 g/Lh lipids.The cost was estimated $1.6/kg lipid, which is more cost-efficient than the eco-certified palm oil. Various parameters were demonstrated that affected the process economics, such as a short fermentation time, solvent-free lipid recovery, and the low cost of acetic acid [8, 16].

Genetic engineering has a key role in flexible optimization of high-value compounds, such as triglycerides, terpenoids, and other metabolically derived value-added compounds, for diverse industrial applications. To date, untargeted genetic manipulation of *C. oleaginosus* has been established using *Agrobacterium*-mediated transformation (AMT) with stable integration of expression cassettes into the genome [17]. However, productivity and product diversity are restricted because of a lack of targeted genome editing. Moreover, the dominance of non-homologous end joining (NHEJ) to homologous recombination (HDR) in *C. oleaginosus* and the lack of an artificial plasmid for this yeast have impeded targeted and efficient genetic engineering [18]. Therefore, the establishment of an efficient and targeted method is necessary for the genetic tractability of *C. oleaginosus* as the most efficient yeast for oil production.

Here, we aim to demonstrate the first targeted genetic engineering method for *C. oleaginosus* using the clustered regularly interspaced short palindromic repeats associated nucleases (CRISPR/Cas) technology. We have established a novel, tailored spheroplasting strategy and transformation method that allows the delivery of all forms of nucleic acids and proteins. Finally, *C. oleaginous* was metabolically engineered to generate various fatty acid (FA) profiles. For this purpose, we selected the Δ-9 and Δ-12 desaturases of *C. oleaginosus* to target the fatty acid metabolism, as they are the key enzymes to synthesize unsaturated fatty acids. Δ-9 desaturase is reported to insert a double bond into saturated fatty acids (such as stearic acid to produce oleic acid). Further, Δ-12 desaturase incorporates another double bond into oleic acid to produce linoleic acid [19, 20].

## Results

### Establishing a method for targeted genetic engineering of *C. oleaginosus*

#### Building a library of guide RNAs (gRNA) for the whole genome of *C. oleaginosus*

For building an *in-silico* library, the whole genome sequence of *C. oleaginosus* was analysed thoroughly. All possible protospacer adjacent motifs (PAMs) for Cas9 and its variants were determined, and the corresponding sgRNAs were added to the library. This enabled the analysis of the off-target activity of each sgRNA against the whole genome of *C. oleaginosus*, which is an essential step for removing and minimising off-target genome integration. However, at present, there are no online tools for the *in-silico* analysis of off-target activities dedicated to *C. oleaginosus*. Therefore, we identified and curated the off-target sites manually.

#### Spheroplast preparation

**Fig. 1:**
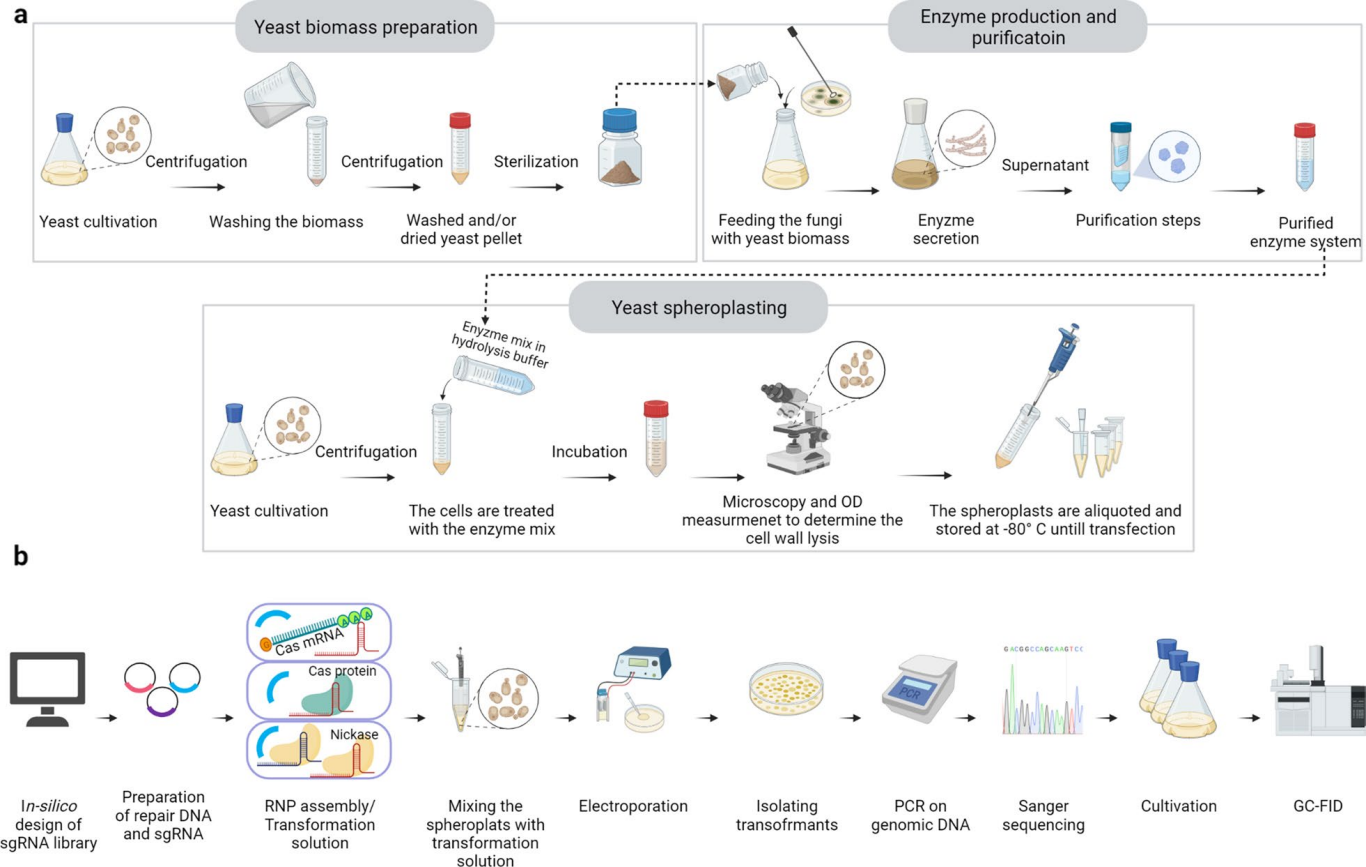
Targeted genetic engineering of *Cutaneotrichosporon oleaginosus* using CRISPR/Cas technology. **a** Schematic illustration of the spheroplasting procedure of *C. oleaginosus* and custom enzyme isolation. **b** Schematic illustration of the genetic engineering of *C. oleaginosus* using CRISPR/CAS system

The self-generated hydrolase enzyme system from *Trichoderma reesei* (HEST) and the commercial Glucanex enzyme resulted in more than 95% of the cells to form spheroplasts after incubation for 15 and 45 min, respectively. This indicates a higher efficiency of HEST owing to the specificity of this enzyme system for *C. oleaginosus* cells (Fig. 1a).

#### Delivery of CRISPR/Cas components into the nucleus

**Fig. 2:**
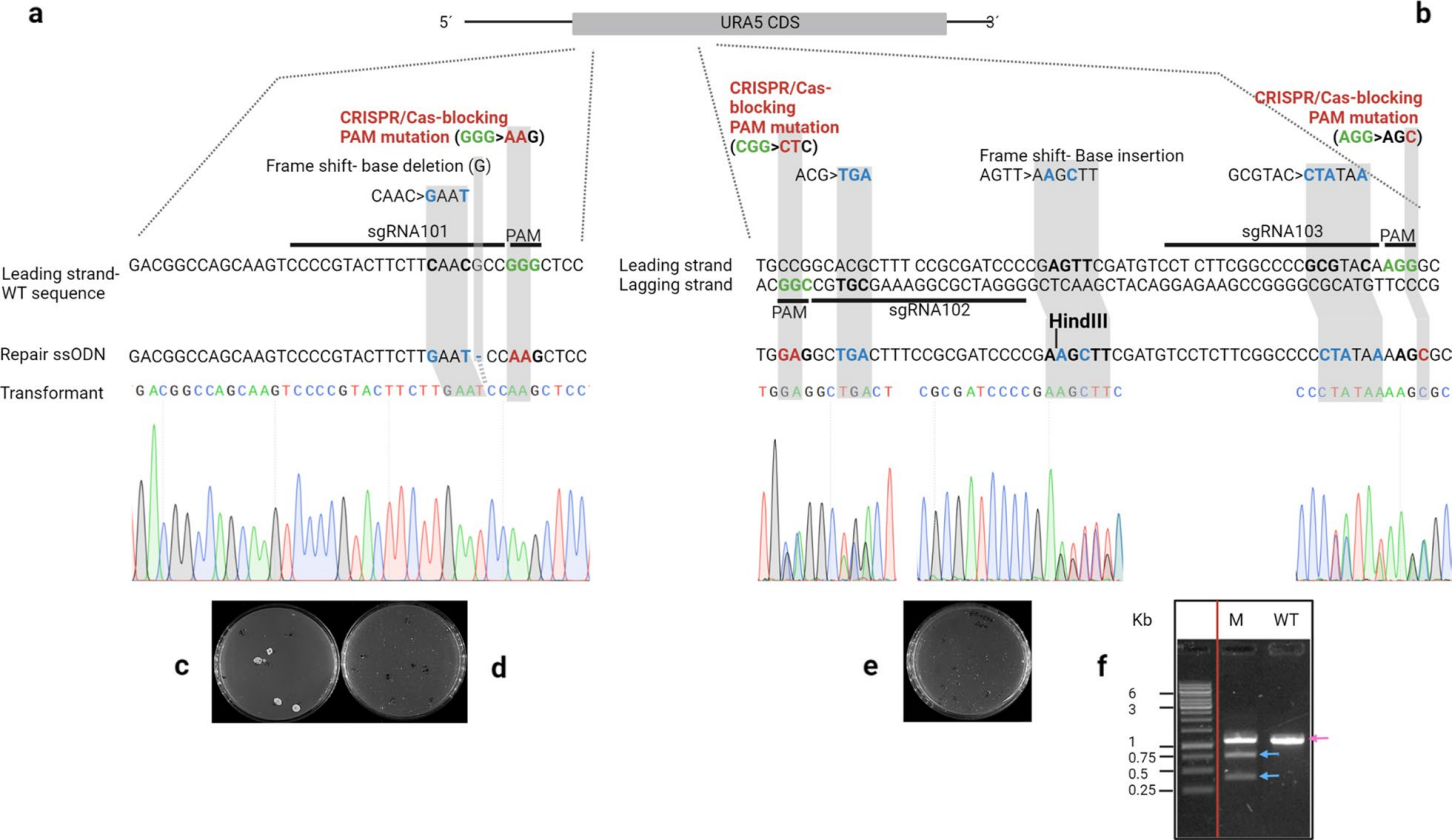
Overview of the *URA5* knockout strategies. As a proof of principle for CRISPR/Cas-mediated genetic modifications, we attempted to knockout the orotate phosphoribosyltransferase gene (*URA5*) to counter select 5-fluoroorotic acid (5FOA). We selected three sgRNAs from the library, which showed no off-target activities within the *C. oleaginosus* genome through *in-silico* prediction, and selectively targeted *URA5*. We then followed three parallel strategies to implement the Cas9 platform in *C. oleaginosus*. Both spheroplast batches prepared by Glucanex and HEST were used to test all strategies. **a** Strategy one and two: genome editing by Cas nuclease delivered into spheroplasts by electroporation in two forms separately: protein (Cas:sgRNA ribonucleoprotein [RNP]) and mRNA. In both strategies one single guide RNA (sgRNA) was used to target *URA5*. A single stranded DNA (ssDNA) was simultaneously transferred to introduce the repair sequences, including base deletions and base substitutions. **b** Strategy three: genome editing using Cas nickase as an RNP. Here, two sgRNAs targeting the leading and lagging strands were delivered to create the double-strand break (DSB). The repair ssDNA included base insertions and substitutions. A non-cutting restriction site (HindIII) was also introduced in the *URA5* loci of mutants. The protospacer adjacent motifs (PAMs) were mutated in all strategies to prevent further DNA cleavage after the repair. **c**–**e** Colonies on selection agar plates with *URA5* knockout using Cas mRNA, nuclease protein, and nickase protein, respectively. **f** The agarose gel electrophoresis of digested *URA5* gene from WT and Δura5 strains. The *URA5* locus was PCR amplified from the genomic DNA of mutants and WT and subjected to fast digestion by HindIII restriction enzyme. The digestion resulted in appearance of two smaller bands in the gene isolated from the Δura5 strain, indicating the integration of repair DNAs by Cas nickase. The WT *URA*5 gene was not digested

The strategies adopted to implement the Cas platform in *C. oleaginosus* are shown in Fig. 2. The delivery of CRISPR/Cas elements into spheroplasts prepared using Glucanex did not generate colonies on the yeast nitrogen base plates containing 5-fluoroorotic acid (YNB^+^5FOA) plates. However, spheroplasts prepared with HEST were successfully transformed. This indicated that the custom-tailored HEST permitted efficient cell wall removal, which allowed for the effective transfer of nucleic acids and proteins into the yeast spheroplasts. Positive clones showed uracil auxotrophy and could not grow in absence of uracil on YNB^−^ plates. Positive transformants were obtained using both the Cas protein and mRNA delivery strategies. The editing efficiency was 100%, 75%, and 20% for Cas9 mRNA, Cas9n D10A protein, and Cas9 protein, respectively. Wild-type spheroplasts were transformed with Cas protein/mRNA and template ssDNA ensured the lack of Cas activity in the absence of sgRNAs. Transformation of spheroplasts with template DNA alone did not produce any colonies.

### Metabolic engineering of *C. oleaginosus* to generate tailored lipid profiles

**Fig. 3:**
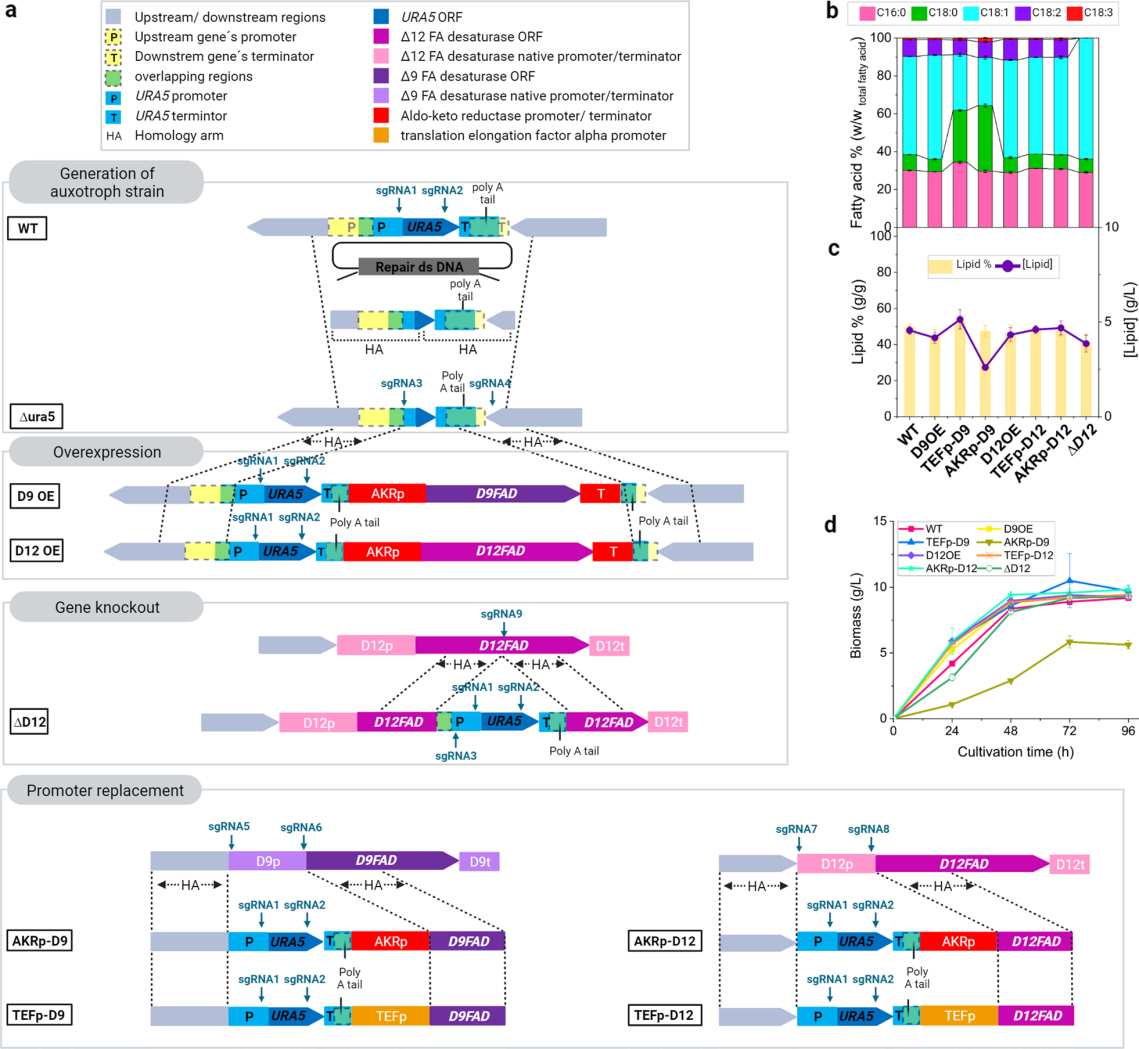
Metabolic engineering. **a** The *URA5* gene, including its promoter, was deleted to generate the Δura5 strain (using single guide RNAs (sgRNAs 1 and 2). The 3’ end of the coding sequence (90 bp) and terminator were not deleted, as they contained the terminator elements of the downstream gene. The complete *URA5* coding sequence, with its native promoter and terminator, was used as a selection marker. The *D9FAD* and *D12FAD* overexpression was accomplished by inserting a second copy fused to AKRp and AKRt, and the selection marker into the upstream region of *URA5* locus in Δ*ura5* strain (sgRNAs 3 and 4), thus generating the strains D9OE and D12OE, respectively. The *D12FAD* knockout was carried out by inserting the *URA5* in the Δ12 desaturase locus (sgRNA9). The *D9FAD* promoter exchange was performed by separate insertion of AKRp or TEFp, and simultaneous deletion of the native promoter to modify their transcriptional regulation (sgRNAs 5 and 6), generating the AKRp-D9 and TEFp-D9 strains, respectively. The same strategy was used for *D12FAD* (sgRNAs 7 and 8), resulting in AKRp-D12 and TEFp-D12, respectively. **b** Fatty acid profile, **c** lipid contents and titres, and **d** growth obtained with the WT and engineered *C. oleaginous* strains in MNM + Glu in shake flasks after 96 h cultivation. All data and error bars represent average and standard deviation of biological triplicates. The WT yielded 9.2 ± 0.2 g/L biomass and 50 ± 1.5% [w_lipid_/dw_biomass_] lipids. The biomass and lipid accumulated by D9OE, D12OE, and TEFp-D9 are comparable to the WT (p > 0.05). In Contrast, the AKRp-D9 exhibited lower growth rate (DCW at 5.6 ± 0.3 g/L) but maintained the cellular lipid accumulation levels after 96 h (47 ± 3% [w_lipid_/dw_biomass_] (p > 0.05)). The *D12FAD* knockout and promoter exchange did not affect the ability of the strains to grow and accumulate lipid

Three approaches were designed for upregulation, downregulation, and knockout of ∆9- and ∆12-fatty acid desaturase genes (*D9FAD* and *D12FAD*) in a uracil-auxotrophic strain (Fig. 3). Targeting *D9FAD* for a full knockout did not result in any transformants with or without oleic acid supply in the YNB^−^ plates.

All strains were analysed under nitrogen limited conditions (MNM + Glu media, Fig. 3). At this stage, the WT FA composition (FAC) was 30% (w/w_total FA (TFA)_) palmitic acid (C16:0), 8% stearic acid (C18:0), 52% oleic acid (C18:1), 9% linoleic acid (C18:2), and 1% linolenic acid (C18:3). Strain D9OE exhibited an increase in C18:1 content (55%) and a decrease in C18:0 content (p < 0.001). Strain D12OE also showed a minimal increase in C18:2 content (p < 0.0001).

Notably, the strains AKRp-D9 and TEFp-D9 displayed significant enhancement of the saturated FAs (S-FAs) (Additional file 2: Table S1; 64% and 62% w/w _TFA_, respectively) at the expense of C18:1 (25% and 29% w/w _TFA_, respectively). This suggests a higher gene expression rate under D9FADp than that under both AKRp and TEFp under the experimental conditions.

Interestingly, the D12FADp replacement caused a slight increase in C18:2 content (p < 0.05), indicating a lower expression rate under the native promoter than both AKRp and TEFp. Furthermore, the knockout of *D12FAD* (∆D12 strain) resulted in the absence of C18:2 and C18:3; consequently, C18:1 content was enhanced by up to 64% w/w _TFA_.

### Tailored lipid production by *C. oleaginosus* using advanced fermentation technologies

**Fig. 4:**
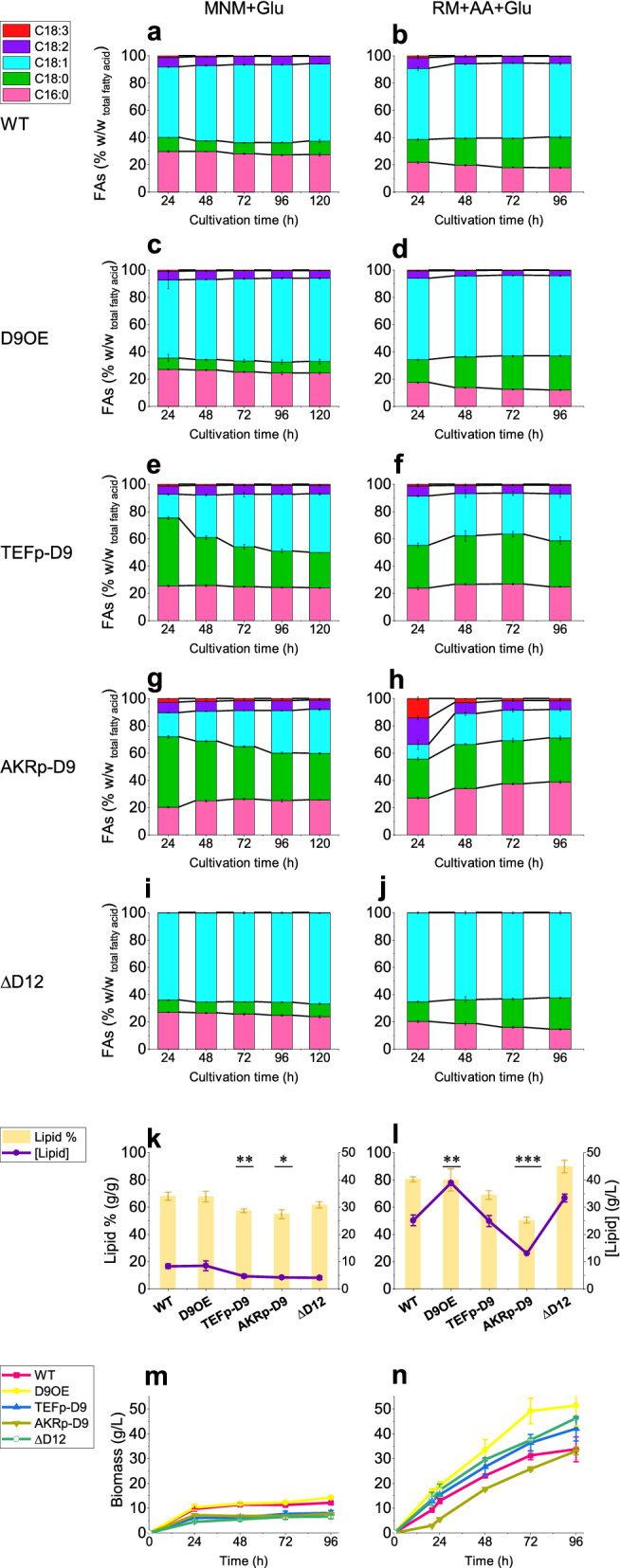
Fed-batch fermentation of *C. oleaginosus* strains. Left column: Fermentation using MNM + Glu. Right column: Fermentation using RM + AA + Glu. **a**, **b** Time course of fatty acid composition (FAC) in WT in MNM + Glu and RM + AA + Glu, respectively. **c**, **d** FAC in D9OE strain. **e**, **f** FAC in TEFp-D9. **g**, **h** FAC in AKRp-D9. **i**, **j** FAC in Δd12. **k**, **l** Lipid contents (yellow bars) and lipid titres (purple line), and **m**, **n** growth (dry cell weights) obtained with the WT and engineered *C. oleaginous* strains in MNM + Glu after 96 h of cultivation and in RM + AA + Glu after 72 h of cultivation, respectively. All data and error bars represent average ± standard deviation of biological triplicates. Statistically significant differences between the WT and each engineered *C. oleaginosus* strain were defined using the two-tailed Student’s *t*-test. * P < 0.05, **P < 0.01, ***P < 0.001, ****P < 0.0001

Selected transformants were subjected to high cell density cultivations using rich medium containing acetic acid and glucose (RM + AA + Glu) and minimal medium (MNM + Glu) (Fig. 4). For the WT, C18:1 content continues to increase slightly throughout the 96 h cultivation (C18:1 content: 57% and 54% w/w _TFA_ in RM + AA + Glu and MNM + Glu, respectively). The final C18:0 yield was higher in RM + AA + Glu than in MNM + Glu (23% and 10% w/w _TFA_, respectively). Moreover, C16:0 was produced in lower ratios in the RM + AA + Glu medium than in the MNM + Glu medium (28% and 18% w/w _TFA_, respectively). The data show that *D9FAD* overexpression has a pulling effect on the elongation of C16:0 towards the production of a higher ratio of C18:1 during fermentation (61% and 59% w/w _TFA_ in RM + AA + Glu and MNM + Glu, respectively). Interestingly, strain D9OE displayed enhanced growth (DCW: 49.2 ± 5 g/L), which in turn resulted in a 54% boost in total lipid titre (38.8 ± 0.8 g/L, p < 0.005) compared to that in the wild-type strain (DCW: 31.3 ± 1.8 g/L; total lipid: 25.1 ± 2 g/L), despite the equal lipid content (Fig. 4 and Table 1). Similar to D9OE, the cultivation of strain ∆D12 in RM + AA + Glu also resulted in a significantly higher total lipid titre (33.3 ± 1.5 g/L, p < 0.05). These data indicate that C18:1 as the main FA in both mutants may enhance yeast biomass production. Similar effects have been reported in the model oleaginous yeast *Yarrowia lipolytica* [21].

**Table 1:** Measurement of lipid content, titre, and biomass productivity

		Lipid						Biomass productivity g/Lh			
		Content % w/w	SD	Titre g/l	SD	Productivity g/Lh	SD	After 96 h	SD	After 72 h	SD
MNM + Glu	WT	67.7	2.84	8.26	0.85	0.11	0.01	0.13	0.01	0.12	0.01
	D9OE	67.5	3.93	8.48	1.79	0.12	0.02	0.15	0.00	0.13	0.00
	TEFp-D9	57.2**	1.36	4.64**	0.62	0.06*	0.01	0.08**	0.01	0.08*	0.01
	AKRp-D9	54.6*	3.26	4.20**	0.12	0.06*	0.00	0.08**	0.00	0.07*	0.00
	ΔD12	61.3	2.20	4.03**	0.65	0.06*	0.01	0.07**	0.01	0.07**	0.01
RM + AA + Glu	WT	80.1	1.82	25.1	1.96	0.35	0.03	0.47	0.07	0.43	0.03
	D9OE	79.6	8.11	38.8**	0.09	0.54**	0.00	0.79*	0.06	0.68*	0.07
	TEFp-D9	68.6*	3.15	24.9	2.04	0.35	0.03	0.58	0.07	0.50	0.05
	AKRp-D9	50.2***	2.04	13.0**	0.14	0.18**	0.00	0.46	0.02	0.36*	0.01
	ΔD12	89.6	4.66	33.3*	1.49	0.41	0.08	0.64*	0.00	0.52	0.01

Lipid analysis was performed after reaching the stationary phase in each medium. In the RM + AA + Glu medium, the analysis was performed after 72 h of cultivation, and in the MNM + Glu medium after 96 h of cultivation in the fed-batch fermentations. The biomass productivity was determined in both media after 72 h and 96 h of cultivation. All data represent averages of biological replicates, and significant differences between the WT strain and each engineered C. oleaginosus strain were defined using the two-tailed Student’s t-test; *P < 0.05, **P < 0.01, ***P < 0.001, ****P < 0.0001

*SD* standard deviation

Interestingly, the promoter substitution of *D9FAD* profoundly affected the FA profile. The comparison of FAC during cultivation highlighted the changes in *D9FAD* regulation. The expression level of *D9FAD* was lower under the control of AKRp and TEFp_,_ resulting in a higher accumulation of C18:0 (TEFp-D9: 50%, 31%; AKRp-D9: 52%, 28% w/w _TFA_) compared with that in the WT (10% and 17% w/w _TFA_) after 24 h in minimal and rich media, respectively. While expression levels under D9FADp varied slightly with the growth phase and media, expression under AKRp and TEFp was strongly correlated with the growth phase and medium. However, the C18:0 content of the TEFp-D9 and AKRp-D9 mutants remained higher than that of the WT throughout cultivation. Specifically, C18:0 content in TEFp-D9 reached 34% and 26% after 96 h in RM + AA + Glu and MNM + Glu, respectively. This implies a lower gene expression level under TEFp control in RM + AA + Glu than in MNM + Glu. Contrary to WT, TEFp-D9 with 24% C16:0, 34% C18:0, and 34% C18:1 yielded sufficient abbre to mimic cocoa butter (CB) FAC, while maintaining a high growth rate and total lipid content. Interestingly, the resulting FAC of AKRp-D9 in RM + AA + Glu showed a notable increase in palmitic acid content (up to 39% w/w _TFA_) after 96 h. Consequently, the unsaturated fatty acids (U-FAs) comprised only 28% of the TFAs, including 21% w/w _TFA_ C18:1 and 7% w/w _TFA_ C18:2, similar to the palm oil FAC. This suggests a lower expression rate under AKRp than under TEFp in the RM + AA + Glu. Compared to WT, the AKRp-D9 strain showed a lower growth rate and total lipid content (Fig. 4), possibly because of higher level of S-FAs [21].

## Discussion

The application of a targeted engineering method without decreasing lipid productivity would improve the significance of *C. oleaginosus* as a flexible source of industrial lipid production. Concerning lipid supply chains, this would allow the transition from an ecologically sensitive, horizontal plant-based agricultural system to a sustainable, vertical cellular agriculture system [22]. Moreover, a precise targeted engineering method helps build a robust platform by enabling an in-depth study of metabolism and gene regulation.

To date, engineering of *C. oleaginosus* has been achieved through untargeted methods, such as untargeted mutagenesis and AMT [17, 23–25], which result in unpredictable gene expression levels, which mainly depend on the variable loci and the number of insertion events. Furthermore, random stable insertion of the *Cas* gene using AMT results in various unwanted genetic alterations. Several reports have demonstrated that *Cas* overexpression results in toxic effects, while continuous and stable expression increases the risk of accumulating off-target effects [26–29]. Further, off-target activity of CRISPR/Cas is reduced when delivered in the form of ribonucleoprotein (RNP) compared with that in case of Cas-expressing gene.

To avoid the aforementioned limitations, we successfully developed an efficient Cas-mediated genome editing technique by employing Cas mRNA and RNP. These two elements permitted circumventing the challenges of promoter efficiency and expression levels of Cas genes. Additionally, it offers DNA-free genome editing. The RNPs are degraded within hours, while the Cas mRNA results in a transient expression of Cas protein, which provides effortless cloning and alleviates off-target effects in eukaryotic cells [30, 31].

Electroporation of *C. oleaginosus* spheroplasts provides a rapid procedure for the delivery of all types of gene elements and proteins. In this study, we established a method for targeted *C. oleaginosus* engineering. Efficient spheroplast isolation was achieved by treating yeast cells with an enzyme mixture isolated from the hydrolase-producing fungus *T. reesei*. Using this tailored enzyme system for *C. oleaginosus*, we successfully removed the cell wall and generated spheroplasts amenable to the transfer of all types of genetic elements and proteins. This was not possible using previously available commercial enzymes. Moreover, spheroplasts can be stored at − 80 °C for extended duration to allow staggered transformations. Cell wall removal is a vital step in this process, where the effectiveness and specificity of the enzyme system plays an essential role. Glucanex, a commercial enzyme commonly used for yeast cell wall removal [32], was ineffective against *C. oleaginosus*, and the transfer of all types of nucleotides and proteins was unsuccessful in the resulting spheroplasts. To date, genetic modifications by transferring Cas9 mRNA for genetic modifications have only been performed in higher eukaryotic cells. Our study, for the first time, shows that genome engineering of eukaryotic microbial cells can be accomplished using Cas9 mRNA.

The GC (61%) rich genome of *C. oleaginous* complicates sgRNA design [18]. While the 20 bp sgRNA is believed to specify the Cas9 for targeted sequence, it was previously observed that three to five mismatches in the PAM-distal part can be tolerated [33]. In addition to PAM, the 10 to 12 bp seed sequence of sgRNA located adjacent to PAM, was suggested to generally plays a more important role in on-target binding of the Cas9:sgRNA complex than the rest of gRNA [34]. Therefore, the existence of similar repetitive sequences in the genome of *C. oleaginosus* contributes to the off-target effects. The Cas9 nickases (Cas9n), which require two adjacent sgRNAs for double strand break generation, decrease off-target effects [35], therefore, facilitate sgRNA design in *C. oleaginosus*. For the first time, nuclease Cas and nickase Cas, which require one and two guide RNAs, respectively, have been successfully incorporated in *C. oleaginosus* by our strategy. A gRNA library specific for *C. oleaginosus* enabled us to eliminate off-targets prior to transformation. The current library has been designed for Cas9 and its variants that require an NGG PAM. However, this can be extended to a variety of PAM sequences targeted by other Cas variants to further increase the availability of PAM options for any target locus.

This study demonstrates the construction of a series of strains with tailored FAC while maintaining a high lipid content, which can be useful for various industrial applications. Our analysis revealed that FAC with a higher oleic acid content favours higher lipid productivity. Compared to that in WT, lipid productivity increased by 54% and 16% in D9OE and ∆D12, respectively. Therefore, applying the D9OE strain to the high-performance process of lipid production reported for *C. oleaginosus* [8] would yield 3.6 g/Lh lipids. In addition to total lipid yield, lipid composition is of industrial relevance. High-oleic oils can be used in the development of bio-lubricants, hydraulic fluids, and oils for electrical transformers because of their excellent oxidative and thermal stability [4]. Moreover, a high monosaturated FA content without PUFAs accumulation (similar to ∆D12) results in an even higher oxidative stability, which can offer a wider temperature range for various applications [36, 37]. Accordingly, the absence of the two PUFAs (C18:2 and C18:3) in ∆D12 resulted in higher oxidative stability of the oil (Additional File 2: Table S1). Furthermore, C18:1 can be channelled into the production of other valuable compounds such as 10-(R)-hydroxy stearic acid [38, 39] by expressing additional enzymes in the respective strains.

Since 1980, *C. oleaginosus* is being extensively investigated by researchers, namely, in an attempt to increase the C18:0 content in yeast oil to produce CB equivalents by decreasing the oxygen supply [40], random mutagenesis [25], or addition of ∆9-desaturase inhibitor [41]. It was reported that *C. oleaginosus* produces higher amounts of TAGs and POP (C16:0–C18:1–C16:0) than that produced by the other oleaginous yeast strains, which makes this strain a potential source for CB-like lipid (CBL) production, when the C18:0 content is increased [42]. We compared the effects of the *D9FAD* native promoter with those of endogenous TEFp and AKRp. The high levels of C18:1 in *C. oleaginosus* WT due to the high expression levels of *D9FAD* were fine-tuned in the TEFp-D9 strain to resemble the CB profile (iodine value: 44, Additional File 2: Table S1,). Currently, stearic acid is the only material used to produce safe solid fats owing to its neutral effect on serum lipoprotein cholesterol. Additionally, highly saturated oil decreases the need for hydrogenation in food and cosmetic products. Genetically improved sunflowers, for instance, produce elevated amounts of stearic and oleic acid. In contrast, WT sunflower oil is mainly composed of U-FAs, which are liquid at room temperature. Therefore, hydrogenation or transesterification is required for solidification [43, 44].

The saturation was further increased in AKRp-D9 cultivated in RM + AA + Glu, similar to the palm oil composition. It implies that *C. oleaginosus* can also accumulate high-saturation oils (72% S-FAs in AKRp-D9) during growth. Moreover, further optimisation would lead to the creation of a strain which can produce FAs with higher contents of both palmitic and oleic acids, equivalent to those in palm oil.

Demand for plant oils in the food, chemical, and pharmaceutical industries is increasing. However, use of tropical plant oils, such as monocrop palm and cocoa, result in tropical forest destruction and adversely affect biodiversity [43, 45]. Among plant oils, palm oil has the highest market volume (market volume: 73 MT in 2020/21 [46]); January 2022: U.S. $1344/ton [47]), and CB is the most highly priced raw material (more than $5000/ton in 2021 [48]; 0.172 MT CB is produced mainly by three countries [49] with wide applications in the food, chemical, and cosmetic industries. Moreover, high-oleic sunflower oil accounts for 32.94% of the global sunflower oil market [50] market volume: 18.46 MT in 2020/21; $2361/ton in March 2022). The supply and climate impact concerns have increased a customer-driven demand for alternative sources, such as yeast oil, which demonstrate an improved ecological profile. The mutants developed in our study demonstrate potential to address the industry demands and concerns.

## Conclusions

In summary, this is the first report demonstrating the targeted engineering of *C. oleaginosus*. The current study unlocks the significant potential for harnessing *C. oleaginosus* for the production of a wide range of oleochemicals for industrial and academic purposes. Optimised pathways lead to a superior cell factory capable of both high productivity and product diversity. Notably, a series of strains with tailored FAC were constructed. Interestingly, the D9OE strain with overexpressed ∆9-desaturase showed elevated C18:1 content and 54% increase in the lipid yield. Further, the ∆D12-desaturase gene knockout resulted in increased C18:1 content and absence of PUFAs in the yeast oil. Furthermore, the CRISPR-mediated genetic engineering using Cas mRNA delivered to yeast spheroplasts were reported for the first time.

The genetic engineering technique developed in our study offers the opportunity to target further strain improvements, including broader substrate utilisation capability, high tolerance to toxic compounds, tailor-made lipids with desired composition or function. Moreover, this novel approach applied to *C. oleaginosus*, coupled with recent advancements, such as the biorefinery process chain including recycling steps [8] and automatable Nile Red analysis [23], can accelerate the creation of a commercially viable, flexible and robust platform. Furthermore, the procedure of the tailored enzyme system isolation for spheroplasting and delivery of Cas elements by electroporation were crucial steps of this flexible technique, which also helps unravelling the genetic accessibility in other unconventional yeasts.

## Methods

### Strain, media, and chemicals

The *C. oleaginosus* ATCC 20509 (DSM-11815) was obtained from the Deutsche Sammlung von Mikroorganismen und Zellkulturen (DMSZ) (Braunschweig, Germany). The wild-type strain was inoculated and maintained on yeast extract peptone dextrose (YPD) agar plates. The auxotroph mutants were maintained on YNB^+^5FOA agar plates (yeast nitrogen base [YNB], 1.7 g/L; NH_4_SO_4_, 5 g/L; uracil, 50 mg/L; glucose, 20 g/L; agar, 20 g/L; 5-fluoroorotic acid [Fluorochem, Germany], 1 g/L), and the top agar contained 0.1–1 g/L 5FOA. Mutants harbouring an auxotrophy-complementing marker gene (selection marker *URA5*) were transferred and maintained on YNB^−^ agar plates (YNB, 1.7 g/L; NH_4_SO_4_, 5 g/L; glucose, 20 g/L; agar, 20 g/L). The top agar plate contained YNB (1.7 g/L), NH_4_SO_4_ (5 g/L), glucose (20 g/L), and agarose (5 g/L). YPD was used as the inoculum medium. For cultivation, two media were used: a minimal nitrogen medium containing glucose (MNM + Glu) [17] and nitrogen rich medium containing glucose and acetic acid (RM + AA + Glu). The latter consisted of the following: glucose, 30 g/L; yeast extract, 3 g/L; peptone, 1.5 g/L; (NH_4_)_2_SO_4_, 0.3 g/L; MgSO_4_·7H_2_O, 1.5 g/L; KH_2_PO_4_, 2.4 g/L; Na_2_HPO_4_, 0.91 g/L; CaCl_2_·2H_2_O, 0.22 g/L; ZnSO_4_·7H_2_O, 0.55 µg/L; MnCl_2_·4H_2_O, 22.4 µg/L; CuSO_4_·5H_2_O, 25 µg/L; FeSO_4_·7H_2_O, 25 µg/L; pH 6.5. *Trichoderma reesei* RUT-C30 (ATCC 56,765) was obtained from the American Type Culture Collection. The cultivation medium for *T. reesei* contained 10 g/L yeast extract, 10 g/L glucose, 1.4 g/L NH_4_SO_4_, 2 g/L KH_2_PO_4_, 0.4 g/L CaCl_2_·2H_2_O, 0.3 g/L MgSO_4_·7H_2_O, 1 g/L NaCl, 5 mg/L FeSO_4_·7H_2_O, 3.7 mg/L CoCl_2_.6H_2_O, 1.6 mg/L MnSO_4_.H_2_O, 1.4 mg/L ZnSO_4_·7H_2_O. The *Escherichia coli* DH5α strain was obtained from Merck Millipore and used for cloning and plasmid amplification. DH5α was grown at 37 °C in Luria–Bertani (LB) medium. Transformed *E. coli* was selected on LB agar plates supplemented with kanamycin sulfate at a final concentration of 50 µg /mL (Roth, Germany).

### Generation of targeting and editing constructs

#### Single guide RNAs

An in-silico sgRNA library was constructed by analysing all scaffolds of the whole genome sequence of *C. oleaginosus* [5] in smaller fragments (using Geneious Prime® 2022.0.1 https://www.geneious.com). All possible PAMs for Cas9 and its variants were determined using Geneious and the corresponding sgRNAs were added to the library (Additional File 1: Data File 1). The off-targets against the whole *C. oleaginous* genome were analysed and on-target activities were determined using the target scoring algorithm reported by Doench et al. [51]. The sgRNAs with zero off-targets and possibly high target scores were selected for genetic engineering and ordered from Synthego (USA) and Eurofins Genomics (Germany) (Additional File 2: Table S2).

#### Repair DNAs

The repair DNA sequences were partially amplified from genomic DNA and cloned into pUC vectors harbouring the kanamycin resistance gene or ordered from Twist Biosciences (USA), and further assembled into plasmids for each experiment. Plasmid construction was performed in DH5α cells using restriction digestion (Thermo Fisher Scientific) and ligation with T4 DNA ligase (Thermo Fisher Scientific), following the manufacturer’s instructions. *E. coli* competent cells were transformed by the heat shock method, plated on LB agar plates containing kanamycin, and incubated overnight at 37 °C. Positive colonies were identified using colony polymerase chain reaction (PCR), inoculated into liquid LB medium, and plasmids were isolated using a GeneJET Plasmid Miniprep kit (Thermo Fisher Scientific). Complete repair DNA sequences were linearised by PCR amplification or using fast digest enzymes and resolved by electrophoresis on a 1% agarose gel stained with MIDORI Green Xtra (NIPPON Genetics). Fast digestion reactions were performed according to the manufacturer’s instructions (Thermo Fisher Scientific, Germany). The PCR reactions were performed using Phusion high-fidelity DNA polymerase (Thermo Fisher Scientific) and Phusion GC-Buffer (Thermo Fisher Scientific) according to the manufacturer’s instructions. The PCR products or digested gene fragments were recovered from the gel using a Monarch DNA Gel Extraction Kit (NEB) and confirmed by Sanger sequencing (Eurofins Genomics, Germany). All the repair DNAs constructed in this study were validated by sequencing and are summarised in Additional File 3: Table S3.

#### CRISPR-Cas enzymes

For synthesising Cas9 mRNA, the *Cas9* gene (including nuclear localisation signal [NLS]) was amplified using Q5 high-fidelity DNA polymerase (NEB) to introduce the T7 promoter upstream of the gene. The quality of the PCR was analysed by agarose gel electrophoresis. The PCR product was purified and used for in vitro transcription using a HiScribe T7 Quick High Yield RNA Synthesis Kit (NEB). RNA was treated with DNase I to remove the DNA template, followed by a spin column purification step (NEB Monarch RNA Cleanup kit). Subsequently, capping (NEB Vaccinia Capping System) and poly A tailing were performed using NEB *E. coli* poly A polymerase. Finally, the mRNA was purified using Monarch RNA Cleanup kit (NEB) and stored at − 80 °C until use. Cas9 enzyme (EnGen Spy Cas9 NLS) and EnGen Spy Cas9 Nickase were purchased from New England BioLabs GmbH (NEB, Germany).

### Strain construction

#### Spheroplasting

A tailored spheroplasting procedure was developed using a previously described method with some modifications [52]. Optimised and efficient spheroplast isolation was achieved by treating the yeast cells with an enzyme mixture isolated from a hydrolase-producing organism, *T. reesei*. This filamentous fungus was cultivated in a cultivation medium supplemented with *C. oleaginous* biomass at 28 °C for 48 h. The supernatant was separated by centrifugation at 2,000 g for 10 min, filtered through a 0.45 µm filter, concentrated 30-fold, and stored at 4 °C.

A single yeast colony was inoculated into 50 mL of YPD medium. The cells were harvested by centrifugation (1,000 g for 5 min) at a density of 2 × 10^7^ cells/mL and washed once with sterile water and once with 1 M sorbitol. Each lysis enzyme (lysis enzyme from *T. reesei* [HEST] and Glucanex) was mixed separately with the spheroplasting buffer (pH 5.8, 1 M sorbitol, 0.1 M sodium citrate, 10 mM EDTA, 30 mM β-mercaptoethanol), to obtain final concentration of 10% v/v for HEST and 2% w/v for Glucanex. The yeast cells were resuspended in the spheroplast buffer and incubated at 30 °C. The spheroplasting progress was monitored at 15 min intervals, by adding 400 µL of the cells to 100 µL of sodium dodecyl sulfate (final concentration of 1% w/v), followed by measuring the decrease in turbidity (OD_600 nm_). Cells were harvested after more than 90% of the cells were converted to spheroplasts. The pellets were washed multiple times using 1 M sorbitol to completely remove the lysis enzymes. The cells were resuspended in 2 mL STC (1 M sorbitol, 10 mM Tris–HCl [pH 7.5], and 10 mM CaCl_2_) and 2 mL frozen cell storage solution (40% glycerol, 14% dimethyl sulfoxide, 0.2 M mannitol, 0.32 M sucrose, 0.1 M sorbitol, 0.2 M trehalose), and stored at − 80 °C until further use for transformation. Cell viability was tested by plating 50 µL of the cells on agar plates containing 1 M sorbitol. For plating, the spheroplasts were added to 5 mL top agar (containing 1 M sorbitol) and plated on agar plates containing 1 M sorbitol. Spheroplasting efficiency was tested by plating 50 µL of yeast spheroplasts onto non-isotonic plates.

#### Yeast transformation

The spheroplasts were thawed and washed thrice with 1 M sorbitol. Then, they were resuspended in sorbitol containing 5% electroporation buffer (0.3 mM Na_2_HP0_4_, 0.02 mM KH_2_PO_4_, 10% glycerol), and incubated on ice for 5 min. Finally, the cells were pelleted and resuspended in 2 mL of 1 M sorbitol mixed with 10 µL electroporation buffer. For electroporation, 50 µL of the spheroplast suspension was mixed with the CRISPR/Cas elements and repair DNA; further, this mixture was transferred to an electroporation cuvette with a 2 mm gap (Biolab, Germany) and pulsed once at 1500 V (MicroPulser Electroporator, Bio-Rad, Germany) according to Bio-Rad’s instructions The cells were resuspended in 5 mL liquid top agar (below 50 °C), poured onto selective agar plates, and incubated at 28 °C until single colonies were visible. The Δ-9 desaturase gene knockout experiments were performed under four conditions; selection plates supplemented with oleic acid (C18:1) (AppliChem, Germany) incubated at 28 °C; selection plates without C18:1 incubated at 28 °C; selection plates supplemented with C18:1 incubated at 37 °C; selection plates without C18:1 incubated at 37 °C. For all experiments, transformants were streaked on new plates and single colonies were inoculated into the selection medium to grow overnight. The editing efficiency was calculated by dividing the number of the positively confirmed transformants by the total number of clones analysed by PCR. The cultures were centrifuged, and genomic DNA was extracted using a yeast DNA extraction kit (Thermo Fisher Scientific). To analyse genetic modifications, PCR was performed on genomic DNA using genome-specific primers upstream and downstream of the targeted locus and subjected to Sanger sequencing.

### Cultivation

Screening experiments were conducted using 100 mL of liquid MNM in 500 mL baffled shake flasks (120 rpm at 28 °C). Cultivation was initiated by inoculating 100 mL of each medium to an OD_600 nm_ of 0.1. The transformants with significant FA change, exhibiting high-value lipid composition, were subjected to high cell density cultivation in bioreactors under controlled conditions. High cell density cultivation was performed in a DASbox four parallel bioreactor system (Eppendorf, Germany) with a working volume (V) of 150 mL each (starting OD_600 nm_ = 0.5) using RM + AA + Glu. The pH was adjusted to 6.5 using 3 M NaOH or acetic acid (70%–100% (w/w)), according to Masri et al. [8]. For control, the MNM with glucose (MNM + Glu) was used as the sole carbon source, and the pH was adjusted to 6.5 using 3 M NaOH or 3 M HCL. In all bioreactors, the temperature was set to 28 °C. Dissolved oxygen was maintained at 50% by stirring at 350–800 rpm; controlled aeration (8.0–1.5 vvm), oxygen ratio (21%–100%), and pressure (1.25–1.5 bar) were maintained during the experiment. An antifoam agent (Antifoam 204, Merck) was used to prevent foaming (5% v/v solution). All cultivations were performed in triplicate. The samples were collected every 24 h.

### Analysis

#### Gravimetric analysis of biomass and lipids

The dry cell weight (DCW) was analysed by pelleting 2 mL culture and washing the pellets with double distilled water (ddH_2_O), followed by lyophilisation in pre-weighed microtubes for 2 days at − 80 °C and 0.04 mbar (VaCo 5, Zirbus Technology, Germany). The intracellular total lipid was measured by extraction using chloroform methanol mixture according to the protocol described by Bligh and Dyer.[53] The harvested cells were washed with ddH_2_O and destructed and homogenised using a high-pressure homogeniser (Mulsiflex C3, Avestine, Canada). Lipids from the homogenates were extracted using two sequential solvent extractions with Folch solution (2:1 chloroform: methanol mixture) for 2 h and 1 h, respectively. After extraction, the chloroform layer containing yeast lipids was aspirated under a nitrogen stream and the lipid content was weighed. The lipid content percentage, total lipid, and lipid and biomass productivities were calculated according to equations Eqs. 1, 2, 3, and 4 respectively:

1 $$Lipid\, content{ \% }w/w=\frac{w\, obtained\, lipid(g)}{w\, obtained\, dried\, biomass (g)}\times 100$$

2 $$Total\, lipid (lipid\, titer\, g/L)=\frac{w\, lipid\, obtained (g)}{V\, of\, culture\, extracted(L)}$$

3 $$Lipid\, productivity g/Lh=\frac{w\, lipid\, obtained (g)}{V\, of\, culture\, extracted\left(L\right)\times incubation\, time(h)}$$

4 $$Biomass\, productivity g/Lh=\frac{w\, biomass\, obtained (g)}{V\, of\, culture\, extracted\left(L\right)\times incubation\, time(h)}$$

#### Fatty acid analysis

Fatty acids composition of the lipids was analysed according to the modified protocol reported by Griffiths et al. [54]. The lyophilised pellets (2–10 mg) were taken in glass tubes and subjected to automated fatty acid methyl esterification using the Multi-Purpose Sampler MPS robotic from Gerstel. For quantification, 500 µL glyceryl trinonadecanoate (C19:0-TAG, 0.2 mg/mL in GC grade toluol) was added to the tubes prior to esterification as an internal standard, followed by mixing at 1000 rpm for 1 min. Next, 1 mL of 0.5 M sodium methoxide dissolved in methanol was added to each tube, heated up to 80 °C, mixed at 750 rpm for 20 min, and then cooled down to 5 °C. Then, 1 mL of hydrogen chloride-methanol solution (Sigma, Germany) was added to each tube, and heated up to 80 °C, mixed at 750 rpm for 20 min, and then cooled down to 5 °C. Finally, 400 μL ddH_2_O was mixed with the samples at 1000 rpm for 30 s, followed by mixing with 1 mL hexane with shaking three times at 2000 rpm for 20 s in a quickMix device. The tubes were centrifuged for 3 min at 1000 rpm and cooled down to 5 °C; 200 μL sample of the organic phase was transferred to a 1.5 mL vial for analysis using GC-2025 Plus gas chromatograph with flame ionisation detection (GC-FID) (Shimadzu, Japan) [55]. Standardisation was performed using the fatty acid methyl ester marine oil standard.

## Supplementary Information

**Additional file 1.** Data File1.gff

**Additional file 2: Table S1.** The fatty acids content and estimated oil properties based on fatty acid profiles. S-FAs = saturated fatty acids (% w/w total fatty acid), U-FAs = unsaturated fatty acids (% w/w total fatty acid), CN = cetane number, IV = iodine value, HHV = higher heating value (MJ/kg), KV = kinematic viscosity (mm2/s), D = density (g/cm3), SV = saponification value, OS = oxidative stability. The S-FAs and U-FAs contents were determined by gas chromatography described in the methods section. Empirical formulas were used to estimate the lipid properties. All data represent averages. **Table S2.** Single guide RNAs used in the study.

**Additional file 3: Table S3.** Repair DNAs used in the study.

## Data Availability

All data supporting the conclusions of this study are included in this published article (and its Additional files).

## Abbreviations

AMT: Agrobacterium-mediated transformation
NHEJ: Non-homologous end joining
HDR: Homologous recombination
FA: Fatty acid
TFA: Total fatty acid
gRNA: Guide RNA
sgRNAs: Single guide RNAs
PAM: Protospacer adjacent motif
CRISPR: Clustered regularly interspaced short palindromic repeats
Cas: CRISPR-associated proteins
HEST: Hydrolase enzyme system from* T. reesei*
5FOA: 5-Fluoroorotic acid
RNP: Ribonucleoprotein
DSB: Double-strand break
Cas9n: Cas9 nickases
YNB: Yeast nitrogen base
YNB + 5FOA: Yeast nitrogen base media containing uracil and 5FOA
ssDNA: Single stranded DNA
dsDNA: Double stranded DNA
YNB −: YNB media lacking amino acids
YPD: Yeast extract peptone dextrose
WT: Wild-type
FAC: Fatty acid composition
MNM + Glu: Minimal nitrogen medium containing glucose
RM + AA + Glu: Rich media containing acetic acid and glucose
DCW: Dry cell weight
LB: Luria–Bertani
CBL: CB-like lipid
GC-FID: Gas chromatography-flame ionization detection
HA: Homology arm
TAGs: Triglycerides
ddH2O: Double distilled water
S-FAs: Saturated FAs
U-FAs: Unsaturated fatty acids
DMSZ: Deutsche Sammlung von Mikroorganismen und Zellkulturen
PCR: Polymerase chain reaction
