# Supplementary material for: Mastering targeted genome engineering of GC-rich oleaginous yeast for tailored plant oil alternatives for the food and chemical sector
Source: Microb Cell Fact. 2023 Feb 8;22:25. doi: 10.1186/s12934-023-02033-1 (PMC9906925; doi:10.1186/s12934-023-02033-1)
Supplement: Supplementary file 2 — Additional file 2: Table S1. The fatty acids content and estimated oil properties based on fatty acid profiles. S-FAs = saturated fatty acids (% w/w total fatty acid), U-FAs = unsaturated fatty acids (% w/w total fatty acid), CN = cetane number, IV = iodine value, HHV = higher heating value (MJ/kg), KV = kinematic viscosity (mm2/s), D = density (g/cm3), SV = saponification value, OS = oxidative stability. The S-FAs and U-FAs contents were determined by gas chromatography described in the methods section. Empirical formulas were used to estimate the lipid properties. All data represent averages. Table S2. Single guide RNAs used in the study. [file 12934_2023_2033_MOESM2_ESM.pdf]

Supplementary information File 2

Table S1. The fatty acids content and estimated oil properties based on fatty acid profiles. S-FAs = saturated fatty acids (% $w/w_{total\ fatty\ acid}$), U-FAs = unsaturated fatty acids (% $w/w_{total\ fatty\ acid}$), CN = cetane number, IV = iodine value, HHV = higher heating value (MJ/kg), KV = kinematic viscosity (mm^2/s), D = density (g/cm^3), SV = saponification value, OS = oxidative stability. The S-FAs and U-FAs contents were determined by gas chromatography described in the methods section. Empirical formulas were used to estimate the lipid properties[1, 2]. All data represent averages.

strains	S-FAs	U-FAs	CN	IV	HHV	KV	D	SV	OS
**WT**	40.42	59.58	63.10	59.00	39.59	4.16	0.94	201.62	23.86
**D9OE**	37.08	62.92	63.22	60.60	39.63	4.21	0.94	200.40	31.06
**TEFp-D9**	58.75	41.25	65.94	44.01	39.59	4.22	0.92	202.90	19.53
**AKRp-D9**	63.41	36.59	66.91	34.64	39.52	4.16	0.91	205.78	17.29
**ΔD12**	37.60	62.40	64.00	56.16	39.62	4.22	0.94	200.88	3824.03

Table S2. Single guide RNAs used in the study.

crRNA sequence	Number
**GCACCAACCAGAGCUGACGU**	sgRNA1
**UCUUCCCAUCAUCUCCAUCG**	sgRNA2
**GCUGUAGUCGACACACCCAG**	sgRNA3
**GAACACCAUACUGGGCGCUG**	sgRNA4
**AAGAAACCCAAGUGGCACAU**	sgRNA5
**GUGACCGUAUGACCGUGGCA**	sgRNA6
**AGGGUGGCGGCAGACAUUGU**	sgRNA7
**CAACAUGACUCCAUGACCCA**	sgRNA8
**UGGACUCGGAGUAGGCCUGG**	sgRNA9
**UGGAUGCUCAUCAAGCCUCG**	sgRNA10

Supplementary References

1. Patel, A., Arora, N., Sartaj, K., Pruthi, V. & Pruthi, P.A. Sustainable biodiesel production from oleaginous yeasts utilizing hydrolysates of various non-edible lignocellulosic biomasses. *Renewable and Sustainable Energy Reviews* **62**, 836-855 (2016).
2. Sergeeva, Y.E. et al. Calculation of Biodiesel Fuel Characteristics Based on the Fatty Acid Composition of the Lipids of Some Biotechnologically Important Microorganisms. *Applied Biochemistry and Microbiology* **53**, 807-813 (2017).
